# Development of High-Performance Catalytic Ceramic Membrane Microchannel Reactor for Carbon Dioxide Conversion to Methanol

**DOI:** 10.3390/membranes16010045

**Published:** 2026-01-17

**Authors:** Aubaid Ullah, Nur Awanis Hashim, Mohamad Fairus Rabuni, Mohd Usman Mohd Junaidi, Ammar Ahmed, Mustapha Grema Mohammed, Muhammed Sahal Siddique

**Affiliations:** 1 Department of Chemical Engineering, Faculty of Engineering, Universiti Malaya, Kuala Lumpur 50603, Malaysiausmanj@um.edu.my (M.U.M.J.); mmgrema@um.edu.my (M.G.M.);; 2 Sustainable Process Engineering Centre (SPEC), Faculty of Engineering, Universiti Malaya, Kuala Lumpur 50603, Malaysia; 3 Department of Mechanical Engineering, Faculty of Engineering, Universiti Malaya, Kuala Lumpur 50603, Malaysia

**Keywords:** methanol synthesis, CO_2_ conversion, membrane microchannel reactor, catalytic membrane, LTA zeolite

## Abstract

Conversion of carbon dioxide (CO_2_) to methanol in a traditional reactor (TR) with catalytic packed bed faces the challenge of lower reactant conversion due to thermodynamic limitations. On the contrary, membrane reactors selectively remove reaction products, enhancing the conversion, but it is still limited, and existing designs face challenges of structural integrity and scale-up complications. Therefore, for the first time, a ceramic membrane microchannel reactor (CMMR) system was developed with 500 µm deep microchannels, incorporated with catalytic membrane for CO_2_ conversion to methanol. Computational fluid dynamic (CFD) simulations confirmed the uniform flow distribution among the microchannels. A catalytic LTA zeolite membrane was synthesized with thin layer (~45 µm) of Cu-ZnO-Al_2_O_3_ catalyst coating and tested at a temperature of 220 °C and 3.0 MPa pressure. The results showed a significantly higher CO_2_ conversion of 82%, which is approximately 10 times higher than TR and 3 times higher than equilibrium conversion while 1.5 times higher than conventional tubular membrane reactor. Additionally, methanol selectivity and yield were achieved as 51.6% and 42.3%, respectively. The research outputs showed potential of replacing the current industrial process of methanol synthesis, addressing the Sustainable Development Goals of SDG-7, 9, and 13 for clean energy, industry innovation, and climate action, respectively.

## 1. Introduction

Carbon dioxide (CO_2_), being a major constituent of greenhouse gas (GHG), is a key driver for global climate change, largely due to the consumption of fossil fuels [1,2]. To combat the problem, countries decided in the Paris Agreement (2015) to limit the temperature rise to well below 2 °C above pre-industrial levels, while achieving net zero emissions (NZEs) by 2050 with an interim target of 45–55% reduction in net GHG emissions by 2030 [3,4,5]. At the recent, Conference of Parties (COP-29), it was agreed upon to boost financial support for meeting the climate goals with rapid actions [6]. However, beyond reducing emissions, capturing and upcycling existing CO_2_ into valuable fuels, such as methanol, offers a promising pathway to decouple fossil fuels from energy chain, achieving carbon neutrality while generating circular economy [7,8,9].

Methanol is an important product of CO_2_ utilization due to its diverse applications in the power sector as vehicle fuel, energy storage, chemical feedstock, and marine fuel. Being in a liquid state, it is convenient for long-term storage and transportation compared to gaseous fuels like hydrogen and ammonia. Although the global production of methanol reached a record 107 million metric tons in 2021 [10], conventional methods still rely on fossil fuels, which pose a significant risk of damage to the climate [11]. Alternatively, producing methanol from captured CO_2_ and hydrogen derived from renewable energy ensures a sustainable and environmentally friendly process [12,13].

In recent decades, the conversion of captured CO_2_ to methanol has gained considerable maturity driven by advancements in catalytic systems. Traditionally, catalytic packed bed reactors utilizing commercial Cu/ZnO/Al_2_O_3_ (CZA) catalyst have been employed for methanol production. However, the reaction is strongly affected by thermodynamic barrier while facing limitations in reactant conversion. As evident from the methanol synthesis reaction scheme shown in Equation (1), it is exothermic in nature and produces less moles of products than reactants. This implies the favorable reaction conditions as low temperature and high pressure, following the Le-Chatelier principle. However, at low temperatures, the reaction kinetics would be too low to meet the production feasibility while high pressure diminishes the cost effectiveness. These process limitations open doors for research towards efficient synthesis of methanol at high temperatures and low pressure. Continuous removal of product from the reaction environment shifts the reaction equilibrium towards the forward direction, surpassing the thermodynamic barrier. In contrast to conventional packed bed reactors, membrane reactors made it possible to selectively remove the certain reaction product, particularly by-product water, offering simultaneous reaction and separation. Selective removal of water at harsh conditions of methanol synthesis, i.e., 180–250 °C temperature and 30–45 bar pressure, was made possible using ceramic-based membranes such as zeolites. Particularly, Linde type A (LTA) zeolite membranes with 4.1 Å pore size and excellent thermal, mechanical, and chemical stabilities have shown good performance in terms of water selectivity and fluxes due to their hydrophilic nature compared to other zeolites [14]. Due to uniform pore openings of the LTA zeolite membrane (4.1 Å), it readily accommodates water molecules (2.69 Å) while restricting larger species such as methanol. The presence of Na^+^ cations further enhances hydrophilicity, promoting preferential water adsorption and diffusion. The combined influence of molecular-sieving and hydrophilic surface chemistry governs the selectivity of membrane towards water molecules [15].

(1) $${CO}_{2}+3H_{2}\to {CH}_{3}OH+H_{2}O\;{∆H}_{298K}=-49.5\;kJ/mol$$

For example, Yue et al. used tubular zeolite-A membrane coated with an active layer of catalyst and managed to achieve 36.1% CO_2_ conversion with 100% methanol selectivity [14]. Meanwhile, Tian et al. used a Cu–ZnO@LTA membrane reactor for CO_2_ hydrogenation, and under similar operating conditions, they achieved nearly twice as much CO_2_ conversion (49.1%) compared to packed bed reactor (26.2%) [16]. Aimed at increasing the hydrophilicity of membrane, a Si-rich zeolite-A membrane was deployed by Seshimo et al. and achieved a good CO_2_ conversion of 60% [17]. Better membrane geometry using a hollow-fiber LTA zeolite membrane by Li et al. resulted in increased CO_2_ conversion by up to 61.4%, which was about three-fold the traditional reactor conversion (23%) under similar reaction conditions [15]. These studies have shown promising results for using membrane reactors for methanol synthesis, catching researchers’ attention regarding further developments in this field. However, these studies are limited to the lab scale and need extensive research to reach maturity. Furthermore, nearly all previous studies used tubular-type membrane reactors, which have limitations in scaling-up, as they add more resistance to the mass transfer, which plays a crucial role in membrane-assisted reactors [18]. It is therefore imperative to devise innovative membrane rector configurations with the potential to overcome the challenges of existing designs.

Emerging membrane microchannel reactors (MMRs), having a high degree of compactness and providing high throughputs, are strong alternatives to traditional tubular-type membrane reactors [19]. One of the most important parameters of a membrane reactor is the surface area available per unit volume of the reactor (m^2^/m^3^), which is significantly high for microchannel reactors [20,21]. In contrast to large-diameter tubular membrane reactors, micron-sized flow paths in MMRs ensure rapid diffusion of species towards membrane surface, giving high mass transfer rates. Furthermore, capacity scale-up by stacking up the unit cells of MMR, makes it industrially viable option for large-scale operation [22]. With all these features, studies performed so far revealed the use of only metallic and polymeric membranes in microchannel reactors. To the best of our knowledge, no studies were reported using ceramic membranes in microchannel reactors, likely due to the difficulties associated with the sealing of ceramic membrane in the microchannel reactor module.

In this study, a ceramic membrane microchannel reactor (CMMR) assisted with a catalytic membrane was developed for CO_2_ conversion to methanol. Computational fluid dynamic (CFD) simulations were performed to study the fluid flow behavior in microchannels in terms of flow distribution and pressure drop and flow uniformity. A flat-sheet LTA zeolite catalytic membrane was synthesized with a thin layer of catalyst coating to serve in the CMMR. The performance of the reactor module was tested for methanol synthesis from CO_2_ with in-situ selective water removal from reaction environment. The research outputs have proved the LTA zeolite-assisted CMMR to be a promising reactor configuration with significantly high conversion, having potential to replace the existing industrial process for methanol synthesis. Furthermore, our work is in line with SDG-7 for affordable and clean energy, SDG-9 for industry, innovation, and infrastructure, and SDG-13 for climate action.

## 2. Materials and Methods

### 2.1. Ceramic Membrane Microchannel Reactor

#### 2.1.1. Reactor Module

**Figure 1:**
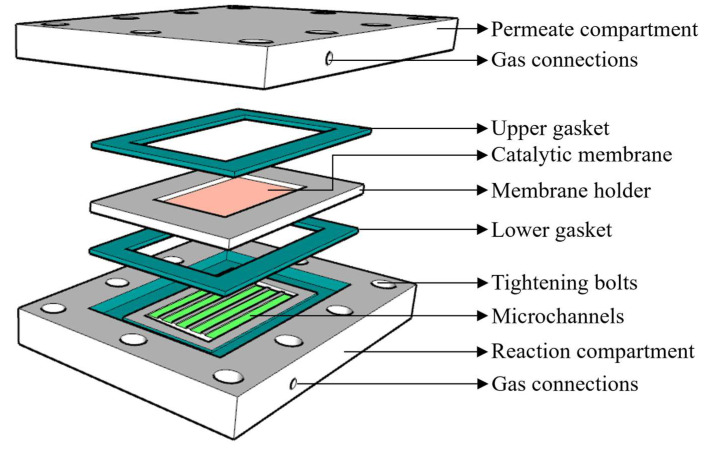
Schematic assembly of ceramic membrane microchannel reactor (CMMR).

The newly designed CMMR module was constructed from stainless steel material, comprising of three major parts, namely, the reaction compartment, membrane holder, and permeate compartment, respectively, from bottom to top, as shown in Figure 1. Each compartment consisted of five 500 µm deep microchannels with dimensions of 5 mm in width and 60 mm in length. Based on our preliminary findings, tapered shaped inlet and outlet headers were constructed to facilitate the uniform distribution of flow in all the channels. In between the two compartments, a membrane holder is sandwiched, which houses the membrane with overall dimensions of 80 mm in length and 43 mm in width; 10 mm width from each long side and 5 mm from each short side of the membrane were consumed for sealing it in the holder using Ceramabond 522 (Aremco, New York, NY, USA), which reduces the effective membrane area to 60 mm × 33 mm. For the sealing of reactor, 2 mm thick Teflon gaskets were used above and below the membrane holder, whereby 1.5 mm deep gasket housing was constructed on each plate, while 0.5 mm thickness of the gasket was provided as compression allowance during the tightening of the reactor. Finally, after stacking all different layers, the overall module was tightened using ten equally spaced bolts.

#### 2.1.2. CFD Simulations

Computational fluid dynamics (CFD) simulations of the microchannel reactor were performed using ANSYS Fluent software (V 19.2) to investigate the flow behavior of gases inside the reactor, assuming a non-reacting, isothermal, constant-pressure, and constant-density system. The aim of the simulations is to isolate the influence of reactor geometry and header design under laminar flow conditions. For this purpose, a full-scale 3D model containing all flow channels with inlet and outlet headers, was constructed in ANSYS Design Modeler (V 19.2). A regular mesh was generated on the entire fluid domain with mesh elements up to 0.25 M, to perform mesh independence study. A simulated fluid with the characteristics of actual feed gas mixture (H_2_:CO_2_ = 3:1) was used for simulations. The flow behavior of the fluid in the reactor was analyzed for different combinations of operating conditions based on the reported literature as 200 °C and 260 °C temperatures, 2.5 and 4.0 MPa pressure, and 10 and 500 mL/min of flow rate, as listed in Table 1. Symbol “Q” was assigned to flow rate, while “C” was assigned to the temperature-pressure combination.

The Reynolds number (Re) of fluid flowing through the reactor was calculated using the following Equation (2).

(2) $$Re=\frac{\rho Dv}{µ}$$

where ρ and µ were the density and dynamic viscosity of gas mixture, respectively, v was the fluid velocity, and D was the hydraulic diameter. The values of Re confirmed the laminar flow for all the combinations of operating conditions. Therefore, a laminar flow model was selected for simulations, which is based on the laws of conservation of mass and momentum, as shown in the following Equations (3) and (4).

Mass conservation (continuity equation):

(3) ∆⋅ν=0

Momentum conservation (Navier–Stokes equation):

(4) $$\rho \left(\frac{\partial \vec{\nu }}{\partial t}+\vec{\nu }\cdot \nabla \vec{\nu }\right)=-\nabla p+\mu \nabla^{2}\vec{\nu }+\vec{F}$$

where $\vec{\nu }$ was the velocity vector, p was pressure, μ was the dynamic viscosity, $\vec{F}$ was the body force such as gravity, and ρ was the density. Fluid velocity at the inlet was given as the initial condition while the no-slip boundary condition was used at the boundary walls. The average velocity at the center of the middle channel was observed for the mesh independence study. Finally, simulation results were obtained in terms of velocity magnitude, pressure drop, and flow distribution in each channel to analyze the flow behavior.

### 2.2. Experimental Procedure

#### 2.2.1. Materials

Chemicals required for the synthesis including tetramethylammonium hydroxide pentahydrate (TMAOH·5H_2_O, CHEMSCENE, Shanghai, China), aluminum iso-propoxide (Al(i-C_3_H_7_O)_3_, Sigma-Aldrich, St. Louis, MO, USA), LUDOX colloidal silica (40 wt.% in water, Sigma-Aldrich, USA), sodium aluminate (NaAlO_2_, Al_2_O_3_: 50–56 wt.%, Na_2_O: 37–45 wt.%, Sigma-Aldrich, USA), sodium hydroxide (NaOH, Merck, Darmstadt, Germany), aluminum nitrate nonahydrate (Al(NO_3_)_3_·9H_2_O, SYSTERM, Shah Alam, Malaysia), zinc nitrate hexahydrate (Zn(NO_3_)_2_·6H_2_O, SYSTERM, Malaysia), copper (II) nitrate trihydrate (Cu(NO_3_)_2_·3H_2_O, Merck, Germany), and sodium carbonate decahydrate (Na_2_CO_3_·10H_2_O, Sigma-Aldrich, USA) were used as received. Deionized (DI) water was used for sample preparation and cleaning purposes throughout the experiments. Flat-sheet porous alumina supports were provided by Lianyungang Baibo New Material Co., Ltd. (Lianyungang, China), with a thickness of 2 mm, pore size of 1–5 μm, and porosity of 40–50% and used after necessary pre-treatment and cleaning.

#### 2.2.2. Synthesis of LTA Zeolite Membrane

The LTA zeolite membrane was synthesized on a flat-sheet macroporous porous alumina support by secondary growth method. Submicron-sized LTA zeolite seeds were hydrothermally synthesized using the gel composition of 1Al_2_O_3_:3.4SiO_2_:0.32Na_2_O:4.2(TMA)_2_O:257H_2_O as per the procedure reported by Li et al. with some modifications [15,23]. Typically, 0.76 g of NaOH, along with 44.8 g of TMAOH·5H_2_O, was added into 102.4 g of deionized water at ambient temperature (25 ± 2 °C) and mixed until full dissolution. Once the solution was clear, 12.0 g of aluminum isopropoxide was added and stirring persisted for an additional 7 h to ensure thorough homogenization. Subsequently, 15.2 g of LUDOX silica was added dropwise, and the resultant suspension was aged for 12 h with continuous agitation. Synthesis was carried out at 100 °C for a duration of 12 h followed by washing with DI water until the pH was reduced to 8. The acquired crystals were subjected to drying in an oven at 100 °C and then calcined at 500 °C for 6 h to eliminate TMAOH.

The flat-sheet macroporous alumina support (length: 80 mm, width: 43 mm) was made ready for the seeding process after surface polishing and washing with DI water and ethanol to remove entrapped debris. Seeding of supports was performed by cross-flow filtration technique with seed loading of 1.5 mg/cm^2^ of support area using a diluted seed suspension of 85 mg/L. Filtration was performed at a flow rate of 8 mL/min, and it was continued until the permeate flow reduced to 10% of the feed with a pressure gradient of 1 bar and then dried at 50 °C overnight.

Hydrothermal synthesis of the LTA zeolite membrane on seeded supports was carried out using a gel composition of 1Al_2_O_3_:5SiO_2_:50Na_2_O:1000H_2_O as per the literature [15,24], with some alterations. Initially, 74.75 g of sodium hydroxide was dissolved in 345.25 g of DI water followed by the addition of 3.8 g of sodium aluminate and continued stirring for 30 min until complete mixing was achieved. Subsequently, 14.8 g of LUDOX silica was added dropwise and the final solution was aged at room temperature for 6 h with continuous mixing. For membrane synthesis, seeded supports were held horizontally in synthesis gel with an inverted position while the top surface (without seeds) was covered with Teflon to avoid crystal growth. Synthesis was carried out at 60 °C for 24 h followed by washing with DI water till the pH had dropped to 8.

#### 2.2.3. Synthesis of Catalyst and Catalytic Membrane

The Cu-ZnO-Al_2_O_3_ (CZA) catalyst with a Cu:Zn:Al ratios of 6:3:1 was synthesized by the co-precipitation method as reported in the literature with some modifications [15]. A 0.2 M solution of copper, zinc, and aluminum nitrates was prepared by mixing the required amounts of salts in deionized water at room temperature. Another 0.2 M solution of Na_2_CO_3_ was also prepared to serve as the precipitating agent. The two solutions were dropped simultaneously in 100 mL of preheated DI water (65–70 °C) with continuous stirring while the dropping rate was regulated to keep the pH of the solution between 7 and 8. The precipitates were aged for 30 min with stirring and then filtered and dried at 110 °C overnight.

A catalyst layer was coated on the LTA zeolite membrane by depositing a thin layer of catalyst precursor on the as-synthesized membrane via the dripping method with catalyst loading of 3 ± 0.1 mg/cm^2^. The required amount of catalyst precursor was dispersed in DI water with a ratio of 1.5 mL of water per mg of catalyst precursor, following 1 h of ultrasonication. The suspension was slowly dropped over the preheated (50 ± 1 °C) membrane placed horizontally. After coating, it was calcined at 360 °C for 4 h, with a heating and cooling rate of 2 °C/min, and the catalytic membrane was ready for further use. The amount of catalyst loaded was confirmed by weighing the dried membrane before and after the entire catalyst loading process.

#### 2.2.4. Performance Test

**Figure 2:**
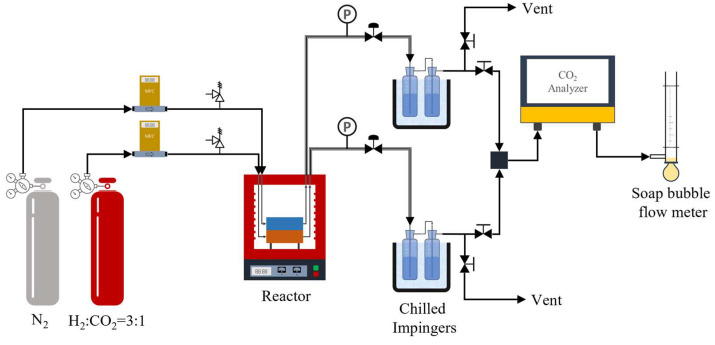
Schematic representation of experimental setup used for testing of reactor.

The performance test of the LTA zeolite catalytic membrane fitted in CMMR was carried out for CO_2_ hydrogenation to methanol. The experimental set-up utilized for this purpose is shown in Figure 2. Mixed gas with the stoichiometric ratio of reactants (H_2_:CO_2_ = 3:1) was fed through a mass flow controller to the reaction chamber. A stream of purified N_2_ gas was supplied to the permeate chamber as sweep gas to reduce partial pressure of permeating species, especially water, thereby stabilizing the membrane performance [25,26]. All gases used in this study were supplied in high-purity form, eliminating the possibility of membrane fouling arising from feed impurities. The reactor module was enclosed in a box-type resistance furnace fitted with a temperature controller. At the exit, both the product and permeate gases were passed through chilled water impinger assemblies to trap methanol while the gas was further sent to the CO_2_ analyzer to estimate the unreacted CO_2_ content. The flow rates of exit gases were measured using the soap bubble flow meter. The methanol content in impinger liquid was measured from gas chromatography (GC, Agilent 6860, Agilent, Santa Clara, CA, USA), fitted with a DB-WAX column and flame ionization detector (FID).

Experiments were performed at 220 °C and 3.0 MPa with a weight hourly space velocity (WHSV) of 18,000 h^−1^, while each experiment was conducted for a duration of 3 h. The performance of CMMR was evaluated in terms of CO_2_ conversion ($X_{CO_{2}}$), CH_3_OH selectivity ($S_{CH_{3}OH}$), and methanol yield ($Y_{CH_{3}OH}$), using the following equations:

(5) $$X_{CO_{2}}=\frac{CO_{2_{in}}-CO_{2_{out}}}{CO_{2_{in}}}\times 100\%$$

(6) $$S_{CH_{3}OH}=\frac{CH_{3}OH_{out}}{CO_{2_{in}}-CO_{2_{iout}}}\times 100\%$$

(7) $$Y_{CH_{3}OH}=\frac{CH_{3}OH_{out}}{CO_{2_{in}}}$$

where $X_{{CO}_{2}}$ is CO_2_ conversion, ${CO}_{2_{in}}$ and ${CO}_{2_{out}}$ are the amounts of CO_2_ in the feed and product streams, respectively, and ${{CH}_{3}OH}_{out}$ is the amount of methanol in the product stream. The results are presented as the average value of three measurements, and standard deviation (SD) is shown as error bars (±SD) on the bar chart. A scaling factor of 20 was applied to SD values to improve their visibility in the bar chart.

### 2.3. Membrane and Catalyst Characterization

Field emission scanning electron microscopy (FESEM) was performed using ZEISS, Gemini Auriga, Oberkochen, Germany, at 2.0 kV to examine the morphology of the synthesized LTA zeolite membrane and the CZA catalyst. Energy-dispersive X-ray spectroscopy (EDS) was performed using AMETEX, EDAX, Mahwah, NJ, USA, at 15.0 kV to characterize the catalyst composition and qualitative assessment of its coating on membrane surface. The elemental composition of prepared catalyst was also determined by X-ray fluorescence (XRF) using the Rigaku Supermini 200, Rigaku Corporation, Akishima-shi, Japan. The X-ray diffraction (XRD) technique was used to determine the crystallinity and phase purity of the LTA zeolite membrane and CZA catalyst using the Rigaku Miniflex, Rigaku Corporation, Japan. The scan was performed using Cu-Kα radiations of wavelength λ = 1.5406 Å, a two-theta (2θ) step size of 0.02°, and a scan speed of 2°/min. N_2_ physisorption at −196 °C was studied on a Micromeritics ASAP2020 Tristar II 3020 Kr, USA instrument (Norcross, GA, USA), to determine the textural properties of the catalyst using approximately 100 mg of sample. The Brunauer–Emmett–Teller (BET) method was applied to determine the specific surface area of the prepared catalyst at a relative pressure of P/P_o_ = 0.99, whereas pore size distribution was calculated by the Barrett–Joyner–Halenda (BJH) method. Hydrogen temperature programmed reduction (H_2_-TPR) analysis was performed for the catalyst using a gas mixture of 5% H_2_ in N_2_ with a flow rate of 10 cm^3^/min and in the temperature range of 50–700 °C with a heating rate of 10 °C/min, using Thermo Finnigan, TPDRO 1100, Waltham, MA, USA. The reduction process was recorded using a thermal conductivity detector (TCD) to measure hydrogen consumption in real time.

## 3. Results and Discussion

### 3.1. Results of CFD Simulations

**Figure 3:**
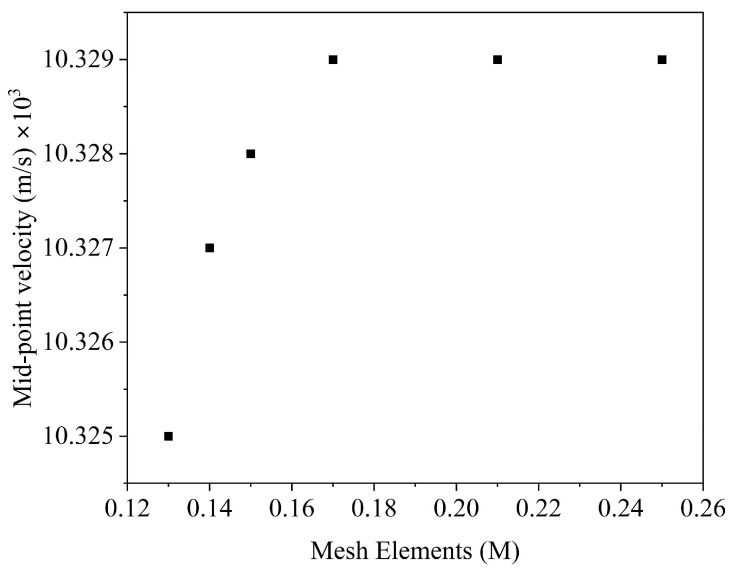
Mesh independence study showing the effect of mesh elements on the mid-point velocity of the middle channel.

CFD simulations were performed to analyze the flow behavior of fluid among different channels. A regular mesh was generated over the entire fluid, and stepwise refining was performed from 0.13 M to 0.25 M elements. The mid-point velocity of the middle channel was observed to perform the mesh independent study, and the results are shown in Figure 3, where it is shown that the velocity magnitude became stable upon further refining the mesh after 0.17 M elements.

**Figure 4:**
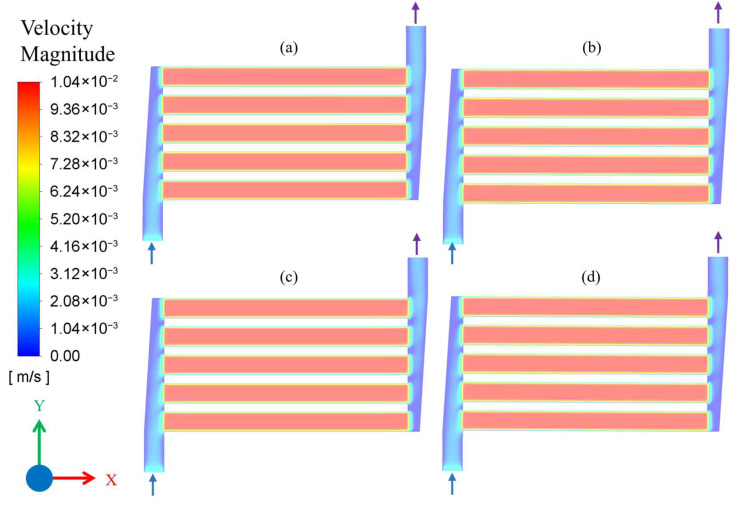
Velocity contour plot of microchannel reactor for flow conditions: (**a**) Q_1_C_1_, (**b**) Q_1_C_2_, (**c**) Q_1_C_3_, and (**d**) Q_1_C_4_. The arrows attached to flow headers indicate the inlet and outlet flow directions.

To analyze the flow behavior, fluid velocity contour plots were obtained over the plane drawn in the middle of flow channels, 0.25 mm above the base. Figure 4 shows the velocity contours for the flow rate of 10 mL/min (Q_1_) and with different combinations of temperature and pressure, as described in Table 1. It is evident from the velocity profiles that flow was uniformly distributed among all the five channels and maximum velocity obtained in the middle of the channels and remained constant throughout the channel length. Due to small flow rates, the flow becomes fully developed immediately after entering the channel. Additionally, there are no noticeable effects of temperature and pressure on velocity profiles for a constant flow of 10 mL/min in all four cases shown in Figure 4.

**Figure 5:**
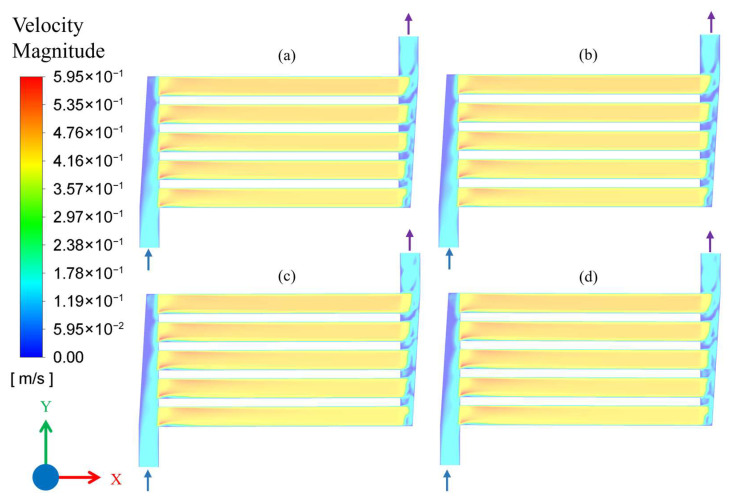
Velocity contour plot of microchannel reactor for flow conditions: (**a**) Q_2_C_1_, (**b**) Q_2_C_2_, (**c**) Q_2_C_3_, and (**d**) Q_2_C_4_. The arrows attached to flow headers indicate the inlet and outlet flow directions.

Consequently, for an increased flow rate of 500 mL/min (Q_2_), the flow behavior was slightly changed from Q_1_, as shown by the velocity contour plot in Figure 5. Performing CFD simulation for such higher flow rates enables a clearer visualization of velocity fields, pressure gradients, and flow uniformity within the microchannel, which is essential for assessing the hydrodynamic performance of the reactor under high-throughput conditions and ensuring stable flow behaviour across a wide operating range. Like the case of Q_1_, the flow is uniformly distributed among the channels, and the effect of temperature and pressure is not significant in all four combinations, as shown in Figure 5. However, the entry effect of microchannels can be observed for the increased flow rate scenario. When fluid enters the microchannels and changes the direction from vertical to horizontal, higher velocity is observed near the bottom of the entrance, while the boundary layer at bottom wall is also relatively thicker, shown with blue shade. As the fluid moves downstream, the boundary layer grows further, making the entry effects vanish, and the velocity profile becomes fully developed in nearly 10% of the total channel length.

**Figure 6:**
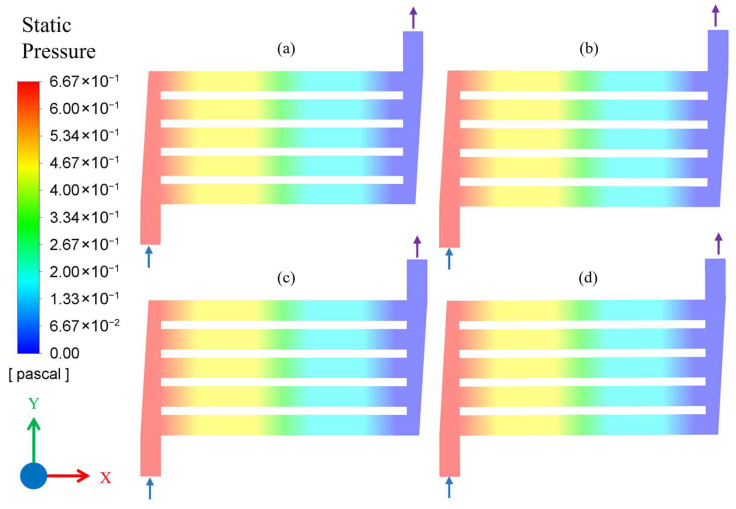
Static pressure contour plot of microchannel reactor for flow conditions: (**a**) Q_1_C_1_, (**b**) Q_1_C_2_, (**c**) Q_1_C_3_, and (**d**) Q_1_C_4_. The arrows attached to flow headers indicate the inlet and outlet flow directions.

Figure 6 shows the contour plots of static pressure over the entire flow area for the Q_1_ flow rate. Due to the small flow rate, the flow was fully developed, and a small pressure drop of nearly 0.5 Pa was observed in each single channel. Furthermore, a gradual pressure drop was seen when flow entered the microchannel from the inlet header. However, the pressure drop behavior for Q_1_ flow rate was observed to be identical for all four combinations of temperature and pressure, as shown in Figure 6.

**Figure 7:**
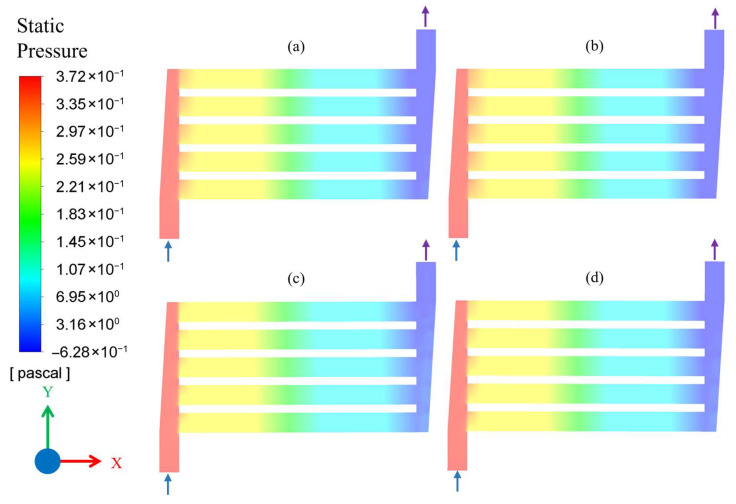
Static pressure contour plot of microchannel reactor for flow conditions: (**a**) Q_2_C_1_, (**b**) Q_2_C_2_, (**c**) Q_2_C_3_, and (**d**) Q_2_C_4_. The arrows attached to flow headers indicate the inlet and outlet flow directions.

For the increased flow rate of Q_2_, a relatively higher-pressure drop of nearly 30 Pa was observed for each single channel. Although the pressure drop of ~30 Pa/channel seems very low compared to conventional catalytic packed bed reactors, it is fully consistent with the geometry and laminar flow regime of the microchannel system in the present study. Unlike the Q_1_ scenario, the entry effect was different for different combinations of temperature and pressure. In Figure 7a,b, for conditions Q_2_C_1_ and Q_2_C_2_, respectively, the entry effects of pressure drop are nearly identical. In contrast, for Figure 7c, corresponding to conditions Q_2_C_3_, there is a sudden pressure drop upon entering the microchannel, which is probably due to the viscous effect corresponding to high pressure (4.0 MPa) and low temperature (200 °C). However, for Q_2_C_4_, shown in Figure 7d, the pressure drop was again relatively more gradual, likely due to less viscous effects appearing at the higher temperature of 260 °C.

**Figure 8:**
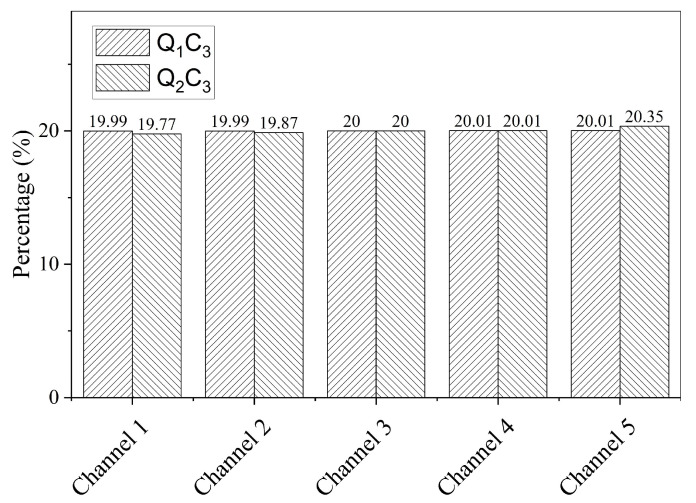
Flow distribution in microchannels for 200 °C temperature and 4.0 MPa pressure with Q_1_ and Q_2_ flow rates.

The flow distribution in microchannels corresponding to different flow rates of Q_1_ and Q_2_ are shown in Figure 8 for C_3_ conditions of 200 °C temperature and 4.0 MPa pressure. For a small flow rate, the flow was almost uniformly distributed among all the channels nearly equal to 20% of flow per channel. On the other hand, a slight variation in flow distribution was observed for Q_2_ flow rate, where bottom channels had to some extent less flow compared to top channels. This is probably due to dominant vertical velocity vectors corresponding to high flow as fluid enters the inlet header. However, the maximum non-uniformity was observed to be less than 1%, which did not have any prominent effect on flow segregation.

Overall, the CFD simulations results revealed the efficient performance of microchannel reactor design while working in milder and harsh conditions of temperature, pressure, and flow rate. Owing to the tapered design of inlet and outlet headers, the fluid was uniformly distributed among all the channels, even working with high flow rates. It is important to mention here that CFD results represent an isothermal, non-reacting baseline studies for quantitatively analyzing flow distribution in the microchannel system.

### 3.2. Results of Synthesized LTA Zeolite Membrane

**Figure 9:**
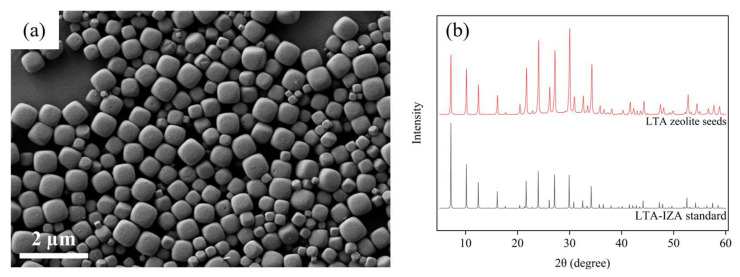
(**a**) FESEM image and (**b**) XRD pattern matching with LTA-IZA standard for submicron-sized LTA zeolite seeds prepared by hydrothermal synthesis.

The LTA zeolite membrane was synthesized on a commercial porous α-alumina support with a pore size in the range of 1–5 µm and porosity of 40–45%, as reported by the manufacturer. The surface morphology of the support used can be seen in the FESEM image shown in Figure S1. Owing to the large pore size of the alumina support compared to that of the water molecule, it offers minimal resistance to the permeation of water molecules. Following the secondary growth method of LTA zeolite membrane synthesis, initially, LTA zeolite seeds were hydrothermally synthesized with a controlled size ranging from 400 to 600 nm (Figure 9a), corresponding to the 12 h of synthesis as reported in the literature [15]. From the FESEM image presented in Figure 9a, the cubical morphology of zeolite seeds confirmed the presence of LTA phase. Furthermore, the XRD analysis shown in Figure 9b also ensures the presence of LTA phase as the pattern of seed samples perfectly aligns with the International Zeolite Association (IZA) database. The presence of LTA phase in seeds is mandatory to achieve phase purity in the selective layer, which was synthesized later.

**Figure 10:**
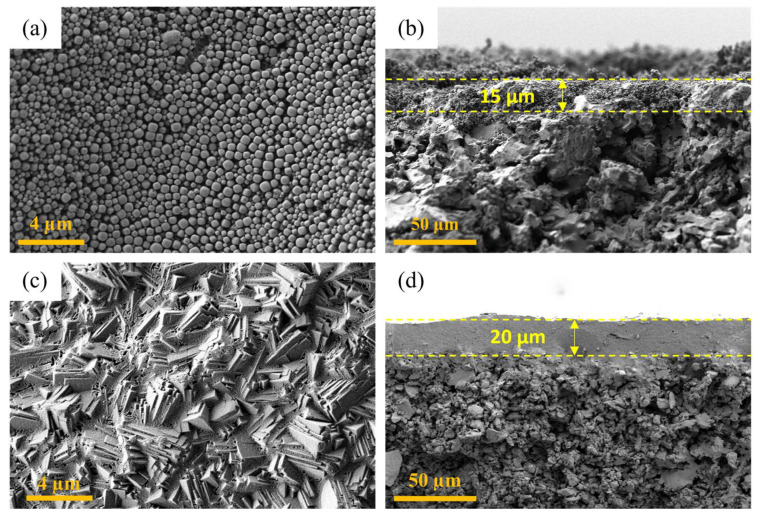
FESEM images of (**a**) surface and (**b**) cross-section of seeded support and (**c**) surface and (**d**) cross-section of LTA zeolite membrane.

For the seeding of α-alumina support, the cross-flow seeding technique was used to obtain a uniform seed layer over the entire surface of the support. As shown in the surface FESEM image of the seeded support (Figure 10a), the presence of a uniform seed layer all over the area confirms the success of the seeding step. Additionally, cross-flow seeding techniques allow the penetration of seeds into the near-surface pores of the support, offering efficient anchoring of the seed layer with the support. Figure 10b shows the cross-sectional view of the seeded support. Owing to the cross-flow filtration seeding method employed in this study, the zeolite seeds penetrated the near-surface pores of the alumina support to a depth of approximately 15 µm, as indicated by the dotted line in the figure. This partial penetration assists in plugging the larger surface pores and provides a more uniform foundation for the subsequent hydrothermal growth, thereby facilitating the formation of a continuous and defect-free LTA zeolite layer, which is difficult to obtain using conventional seeding techniques such as dip coating and rubbing [23,27,28].

**Figure 11:**
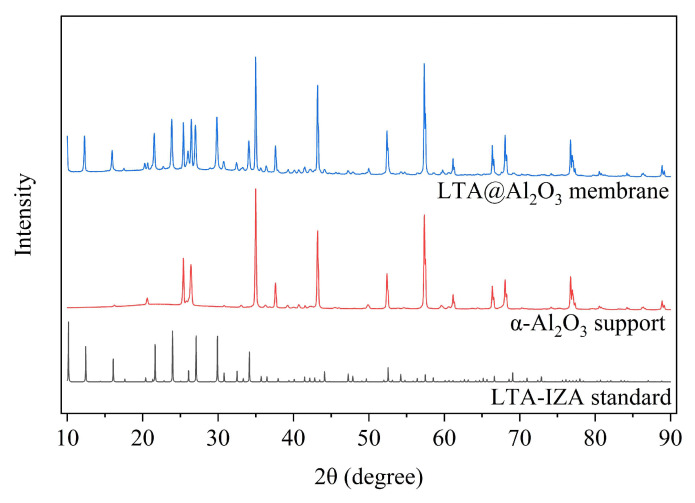
XRD pattern of LTA zeolite membrane synthesized on α-alumina support and mapping with LTA-IZA standard.

Finally, the LTA zeolite layer was synthesized on a seeded support by the hydrothermal method carried out at 60 °C for 24 h. From the surface image presented in Figure 10c, a well-interconnected layer composed of cubical crystals of LTA zeolite can be seen clearly with no visible defects. The thickness of the synthesized LTA zeolite membrane was about 20 µm, shown as a dense layer in Figure 10d, whereas at the membrane–support interface, a good overlapping of the dense and porous layer is present, which was aided by the penetration of seeds into the porous support. Furthermore, in order to confirm the phase purity of the synthesized layer, XRD analysis was performed for the final membrane and the α-alumina support alone, as presented in Figure 11. From the results, it can be clearly seen that the peaks of final LTA zeolite membrane are perfectly matching with LTA zeolite and α-alumina patterns, which confirms the phase purity of the synthesized LTA zeolite membrane. The quality of the synthesized LTA zeolite membrane, as confirmed by FESEM and XRD analysis, is consistent with the previously published literature that demonstrated good permeation properties and successfully employed similar membranes in methanol synthesis environments [15,23].

### 3.3. Catalyst and Catalytic Membrane

The elemental composition of the synthesized catalyst was determined using X-ray fluorescence (XRF) and energy-dispersive X-ray spectroscopy (EDS), and results are presented in Table 2. The expected composition of CZA catalyst composed of Cu, Zn, and Al was 60%, 30%, and 10% respectively, as per the stoichiometry of the metal solution. However, the measured compositions obtained from XRF were 64.1%, 34.4%, and 1.4%, while from EDS, they were 59.2%, 29.2%, and 11.6% for Cu, Zn, and Al, respectively. Such minor deviation between desired and actual composition could arise from inefficiencies during the precursor mixing, drying, or calcination process. The aluminum content was not accurately measured by XRF due to its lower atomic number and the detection limit of the instrument. However, the presence of Al was assured from synthesis stoichiometry and further confirmed by EDS analysis.

Textural properties of the synthesized CZA catalyst were determined by physisorption of N_2_ at −196 °C, and the results are presented in Table S1, which are in good accordance with literature reports [29,30]. The catalyst exhibited a BET surface area (S_BET_) of 79.2 m^2^/g with a pore volume of (V_P_) of 0.5 cm^3^/g. The average pore diameter of the catalyst was measured as 25.2 nm, which clearly lies in the range of mesopores (2–50 nm) [30]. The XRD pattern of the calcined catalyst is displayed in Figure S2, showing distinct diffraction peaks of CuO and ZnO, consistent with the reported literature [30,31,32]. The H_2_-TPR curve of the catalyst is shown in Figure S3, where the presence of a single sharp peak at 253 °C indicates the presence of uniform and well-dispersed CuO phase, which matches with TPR profiles of the CZA catalyst reported in the literature [30,33].

**Figure 12:**
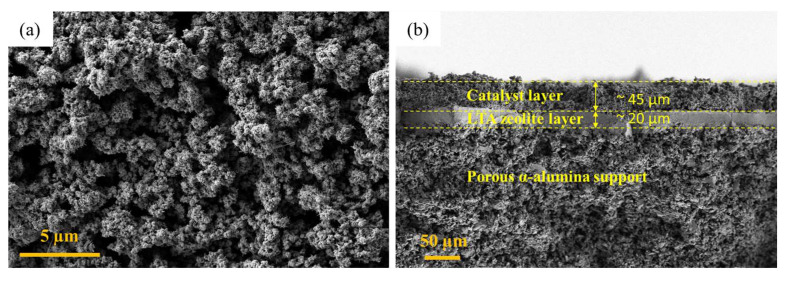
FESEM images of catalytic membrane (**a**) surface and (**b**) cross-section.

A catalytic membrane was prepared by depositing a thin layer of CZA precipitates on the surface of the synthesized LTA zeolite membrane followed by calcination at 360 °C, depositing a uniform layer of catalyst on the membrane surface weighing 42.07 mg. Figure 12 presents the FESEM micrographs of the prepared catalytic membrane. From the surface image (Figure 12a), the crystalline structure and porous morphology of the catalyst layer can be seen clearly. The significant void spaces in the catalyst layer allow the penetration of reactant gases into the layer, ensuring sufficient contact with catalyst particles. Additionally, the porous layer also facilitates the passage of by-product water towards the membrane surface, where it passes through the selective layer of the LTA zeolite. From the cross-sectional image (Figure 12b), a three-layer structure can be seen: catalyst (~45 µm), LTA zeolite membrane (~20 µm), and porous α-alumina support from top to bottom, respectively.

**Figure 13:**
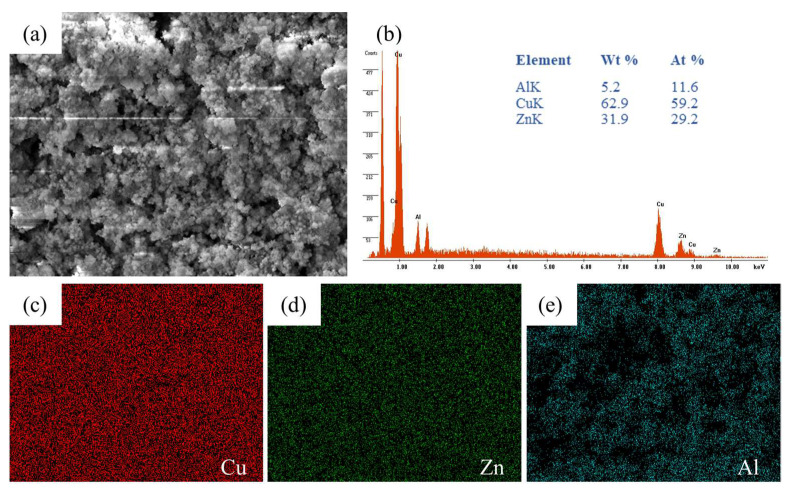
EDS analysis for the surface of the catalytic membrane: (**a**) visual image, (**b**) elemental composition, (**c**) Cu mapping, (**d**) Zn mapping, and (**e**) Al mapping.

Figure 13 presents the EDS results for the top surface of the catalytic membrane including the visual image shown in Figure 13a, elemental composition in Figure 13b, and elemental mapping of Cu, Zn, and Al presented in Figure 13c, Figure 13d, and Figure 13e, respectively. The EDS spectrum obtained from the analysis region showed the major peaks corresponding to Cu, Zn, and Al, as displayed in Figure 13b. The elemental composition, obtained from EDS analysis as Cu (59.2 At. %), Zn (29.2 At. %) and Al (11.6 At. %), is in close agreement with the actual amounts used, as mentioned in Table 2. Furthermore, elemental mapping of Cu, Zn, and Al confirmed a homogeneous distribution over the entire analyzed area, whereas the intensity in each map is proportional to the amount of respective metal present in the sample as Cu > Zn > Al.

Following the development of the optimized microchannel reactor design and the successful incorporation of the catalyst layer on the LTA zeolite membrane, the system must be validated for its performance in CO_2_ conversion to methanol.

### 3.4. Performance Test

**Figure 14:**
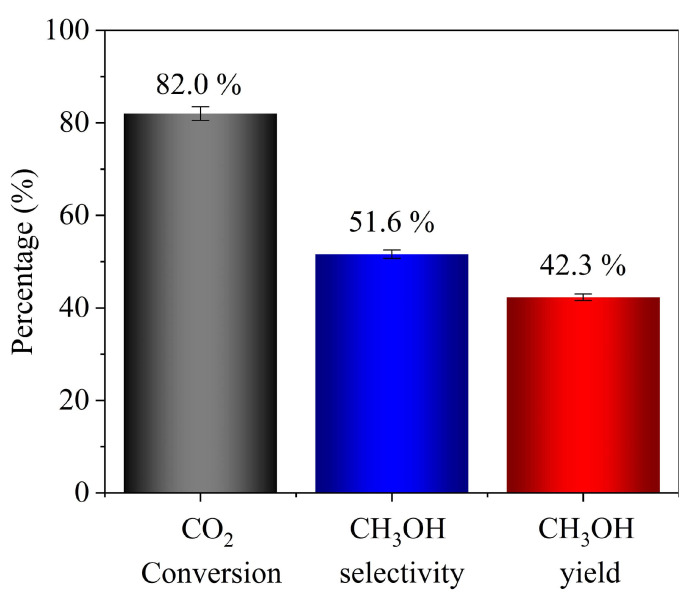
Performance test results of CMMR methanol synthesis experiments.

The performance of the catalytic ceramic membrane microchannel reactor was tested in a methanol synthesis experiment at operating conditions selected to be close to the middle of the CFD simulations range: 3.0 MPa pressure and 220 °C temperature with a WHSV of 18,000 h^−1^. The selected temperature and pressure fall within the conventional methanol synthesis window (180–250 °C, 3–4.5 MPa), where the Cu-ZnO-Al_2_O_3_ catalyst exhibits high stability and minimal carbon deposition, which is mainly associated with CO-rich feed [15,34]. Prior to the catalytic reaction, the catalyst layer was reduced in situ at 253 °C under the flow of 10% H_2_ in N_2_ at ambient pressure for 5 h. The performance of the reactor system was measured in terms of CO_2_ conversion, methanol selectivity, and methanol yield, as shown in Figure 14. During the experiments, no measurable decline in the outlet CO_2_ concentrations was observed, which confirms stable membrane performance. The concentrations of CO_2_ and methanol were quantified in both permeate and retentate streams, and these combined values were used as the primary performance indicators for evaluating membrane-assisted methanol synthesis. These two species were selected because they directly reflect CO_2_ conversion and methanol productivity, which are the key metrics relevant to the feasibility assessment performed in this work. It is worth mentioning that a significantly higher CO_2_ conversion of around 82.0% was achieved in this newly designed CMMR system with 51.6% methanol selectivity and 42.3% yield. The achieved conversion is approximately 3 times higher than equilibrium conversion (27% at 250 °C and 5.0 MPa) and 10 times higher than a traditional packed bed reactor (8.5% at 250 °C and 3.0 MPa), as reported by Makertiharta et al. [35], at comparable operating conditions, listed in Table 3.

The significantly increased CO_2_ conversion was made possible due to the presence of LTA zeolite membrane inside the reactor, which preferably removed the by-product water, shifting the reaction equilibrium towards the forward direction, based on the Le Chatelier principle. The comparison of experimental results with the literature using membrane reactors for methanol synthesis is compiled in Table 3. The CO_2_ conversion obtained in this study is remarkably higher than tubular and hollow-fiber membrane reactors. For example, Yue et al. used a tubular LTA zeolite membrane coated with an active layer of catalyst and managed to achieve 36.1% CO_2_ conversion operating at 260 °C and 3.0 MPa [14], whereas Li et al. achieved further higher CO_2_ conversion of 61.4% operating at 250 °C and 3.5 MPa while utilizing a hollow-fiber membrane design and packed bed of catalyst. Describing the effectiveness of membrane uses in microchannel reactor system, the quantitative assessment of CO_2_ and methanol collected in permeate and retentate sides are mentioned in Figure S4 in Supporting Information. The methanol selectivity of 51.6% obtained in this study refers to methanol formed relative to all products in the reactor effluent from 82% of CO_2_ conversion. The possible side products due to competing side reactions mainly include CO from the reverse water–gas shift (RWGS) reaction and DME from the dehydration of methanol. Numerous studies on CZA catalysts have reported that CO is the primary side product formed via RWGS reaction during CO_2_ hydrogenation to methanol [15,36,37]. However, the analysis of liquid-phase products performed using GC-FID showed only methanol with no detectable traces of other liquid byproducts.

**Table 3:** Performance comparison of CMMR with the data reported in the literature.

Reactor Type	Catalyst	Temperature (°C)	Pressure (MPa)	CO_2_ Conversion	CH_3_OH Selectivity	Reference
Equilibrium conditions	NA *	250	5.0	27	68	[35]
Traditional packed bed reactor	Cu-ZnO-Al_2_O_3_	250	3.0	8.5	33	[35]
Packed bed tubular membrane reactor	Cu-ZnO-Al_2_O_3_	256	2.0	16.5	37.9	[38]
Catalytic membrane tubular reactor	Cu-ZnO-Al_2_O_3_-ZrO_2_	220 260	3.0 3.0	26.5 36.1	93.2 100	[14]
Catalytic membrane tubular reactor	Cu/Zn-BTC	250	3.0	49.1	93.4	[16]
Packed bed hollow-fiber membrane reactor	Cu-ZnO-Al_2_O_3_	220 250	3.5 3.5	57.2 61.4	67 45	[15]
Catalytic membrane microchannel reactor	Cu-ZnO-Al_2_O_3_	220	3.0	82	51.6	This work

* Not applicable.

The higher CO_2_ conversion achieved in this study could be referred to two main influencing factors. Firstly, instead of making a packed bed of catalyst, a thin catalyst (~45 µm) layer was deposited on the membrane surface, as shown in Figure 12b, which greatly increased the exposed surface area of the catalyst, hence increasing the reaction probability. Additionally, due to the reaction occurring at the membrane surface instead of bulk volume, the by-product water produced is subsequently captured by the zeolite membrane present beneath the catalyst layer. Secondly, the microchannel reactor with a channel depth of 500 µm provides a substantially smaller volume compared to conventional tubular designs with a diameter up to 10 mm. The reduced reactor volume favors the reaction equilibrium to shift in the forward direction, as four moles of reactants are converted into two moles of products, thereby enhancing the overall reactant conversion. This equilibrium behavior observed in the microchannel reactor system is consistent with the Le Chatelier principle, which predicts a shift towards the side with fewer moles under reduced volume conditions. Furthermore, due to the small volume of the flow channel, the diffusion length of molecules decreases greatly while reaching the catalyst surface, which ensures frequent and efficient encounters of reactant molecules with the catalyst [22]. Although Yue et al. [14] used a catalytic membrane instead of a packed bed reactor, they could only achieve 36.1% CO_2_ conversion due to the tubular design of the membrane reactor where the diffusion lengths are relatively larger, offering less efficient encounters of reactant gases with the catalyst layer.

The key innovativeness of this work lies in the development of a new type of microchannel reactor with the ability to incorporate a ceramic-based membrane. This design utilized a previously established LTA zeolite membrane in a microchannel reactor, enabling enhanced heat and mass transfer, leading to significantly higher CO_2_ conversion compared to conventional membrane reactor designs such as tubular- and hollow-fiber designs, as listed in Table 3. Furthermore, the microchannel configuration and thin catalyst layer employed in this study minimize local temperature gradients, which further suppresses thermally driven deactivation due to Cu sintering. Collectively, combining the effects of the layered design of catalytic membrane and small-volume microchannels, the efficient conversion of CO_2_ was achieved, representing a breakthrough over the conventional tubular membrane reactor design.

## 4. Conclusions

In this study, a ceramic membrane microchannel reactor was successfully developed, incorporating a catalytic LTA zeolite membrane for efficient conversion of CO_2_. The design of the microchannel reactor was investigated by CFD simulations, which proved the uniform distribution of flow and pressure drop among all the microchannels, owing to the tapered design of inlet and outlet headers. Due to small flow areas, flow was in a laminar regime and the entry effects were observed to be minimum even at high flow rates, which allowed the flow to become fully developed within 10% of the channel length. The membrane reactor design has the advantage of performing reaction and separation simultaneously, which beats the thermodynamic barrier due to the removal of byproduct water from the reactor. In contrast to the catalytic packed bed, coating a thin layer (~45 µm in this study) of catalyst on the membrane surface offers the additional advantage of increased catalyst surface area and subsequent drifting of water molecules towards membrane surface immediately after the reaction. However, coupling the catalytic membrane with the microchannel reactor took further advantage of the small flow volume of reactor, which offered reduced diffusion lengths to the reactant molecules, making encounters of reactant molecules with catalyst particles more efficient, which could have contributed to achieving the higher conversion of 82.0%. Furthermore, the small volume of reactor (0.5 mm deep microchannels) pushes the reaction equilibrium towards the product side, which contains a smaller number of moles compared to the reactant side. In conclusion, the CMMR design gave promising results for CO_2_ conversion to methanol, showing 82.0% CO_2_ conversion, 51.6% methanol selectivity, and 42.3% methanol yield while operating at a temperature of 220 °C and 3.0 MPa pressure. The CO_2_ conversion achieved in this study even surpassed the figures obtained in the conventional tubular design of membrane reactors, a significant value addition to the industrial methanol synthesis process. Findings of the study contributed to meeting several Sustainable Development Goals such as SDG-7 for affordable and clean energy, SDG-9 for industry, innovation, and infrastructure, and SDG-13 for climate action.

## Figures and Tables

**Table 1:** Different combinations of physical properties for flow simulations in microchannel system.

Simulation No.	Flow Rate (mL/min)	Pressure (MPa)	Temperature (°C)
Q_1_C_1_	10	2.5	200
Q_1_C_2_	10	2.5	260
Q_1_C_3_	10	4.0	200
Q_1_C_4_	10	4.0	260
Q_2_C_1_	500	2.5	200
Q_2_C_2_	500	2.5	260
Q_2_C_3_	500	4.0	200
Q_2_C_4_	500	4.0	260

**Table 2:** Composition analysis of synthesized CZA catalyst.

Component	Expected Composition (At. %)	XRF Composition (At. %)	EDS Composition (At. %)
Cu	60	64.1	59.2
Zn	30	34.4	29.2
Al	10	1.4	11.6
Cu/Zn	2	1.9	2.0

## Data Availability

The original data presented in the study are included in the article and Supplementary Materials, further inquiries can be directed to the corresponding authors.

## Supplementary Materials

The following supporting information can be downloaded at: https://www.mdpi.com/article/10.3390/membranes16010045/s1. Figure S1: Surface FESEM image of porous alumina support used for synthesis of LTA zeolite membrane; Figure S2: XRD pattern of calcined CZA catalyst; Figure S3: H_2_-temperature programmed reduction curve for CZA catalyst; Figure S4: Percentage distribution of CO_2_ and methanol in permeate and retentate streams; Table S1: Properties of CZA catalyst determined by N_2_ physisorption at −196 °C. Refs. [30,31,32] are cited in Supplementary Materials.
