# Supplementary material for: Development of High-Performance Catalytic Ceramic Membrane Microchannel Reactor for Carbon Dioxide Conversion to Methanol
Source: Membranes (Basel). 2026 Jan 17;16(1):45. doi: 10.3390/membranes16010045 (PMC12843860; doi:10.3390/membranes16010045)
Supplement: Supplementary file 1 [file membranes-16-00045-s001.zip › membranes-4042884-supplementary.pdf]

Development of High-Performance Catalytic Ceramic Membrane Microchannel Reactor for Carbon Dioxide Conversion to Methanol

Aubaid Ullah [1], N. Awanis Hashim [1,2,*], Mohamad Fairus Rabuni [1,2,*], Mohd Usman Mohd Junaidi [1,2], Ammar Ahmed [3], Mustapha Grema Mohammed [1], Muhammed Sahal Siddique [1]

[1] *Department of Chemical Engineering, Faculty of Engineering, Universiti Malaya, 50603, Kuala Lumpur, Malaysia*
[2] *Sustainable Process Engineering Centre (SPEC), Faculty of Engineering, Universiti Malaya, 50603 Kuala Lumpur, Malaysia*
[3] *Department of Mechanical Engineering, Faculty of Engineering, Universiti Malaya, 50603, Kuala Lumpur, Malaysia*
**Correspondence*: awanis@um.edu.my (N.A.H.); fairus.rabuni@um.edu.my (M.F.R.)

Supplementary Information

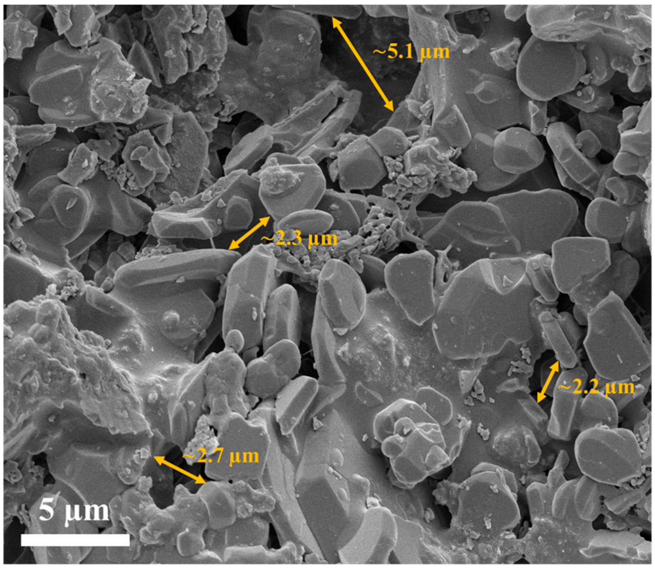

Figure S1: Surface FESEM image of porous alumina support used for synthesis of LTA zeolite membrane

Table S1: Properties of CZA catalyst determined by N_2 physisorption at –196°C

**Property**	**Value**
BET surface area, S_{BET} (m^2/g)	79.2
Pore volume, V_P (cm^3/g)	0.5
Pore diameter, d_P (nm)	25.2

XRD pattern of calcined catalyst is displayed in Figure S1, showing distinct diffraction peaks of CuO at 2θ values of 35.6°, 38.7°, 48.8°, 61.6°, and 66.2°, while for ZnO appearing at 2θ values of 31.7°,36.4°, 56.6°, 62.8° and 68.0°, consistent with the reported literature [1–3].

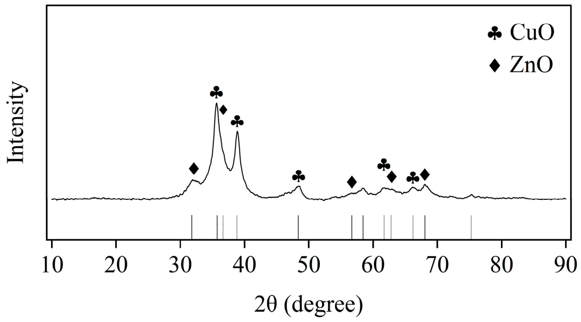

Figure S2: XRD pattern of calcined CZA catalyst

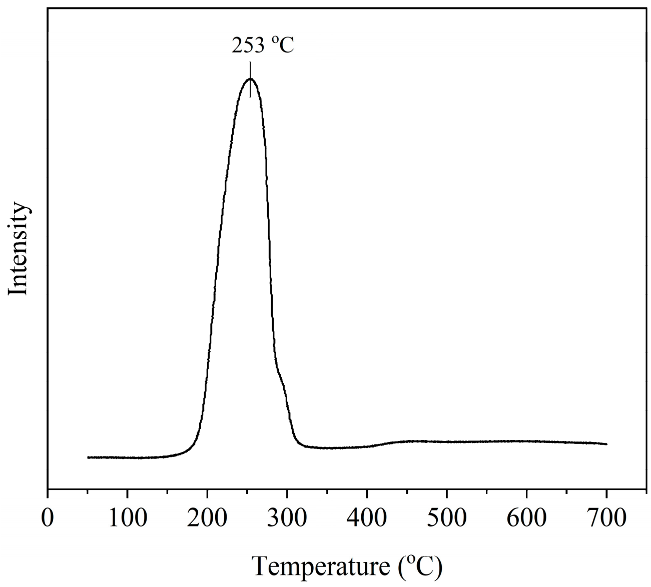

Figure S3: H_2-temperature programmed reduction curve for CZA catalyst.

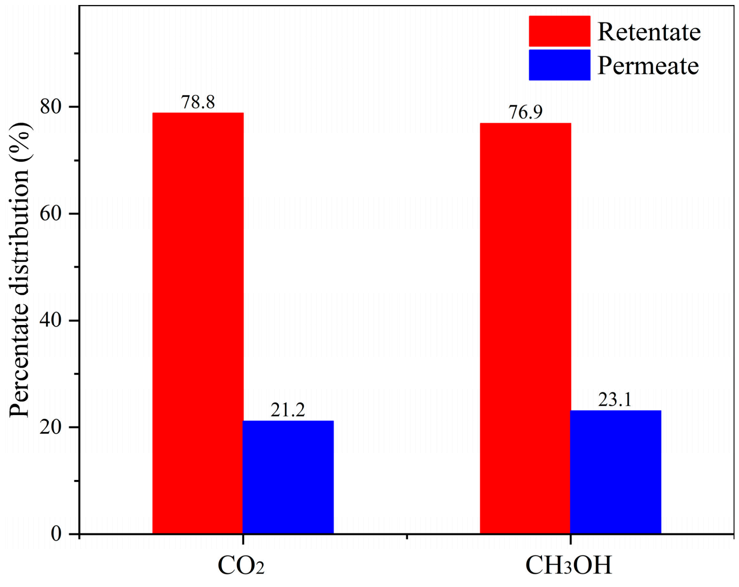

Figure S4: Percentage distribution of CO_2 and methanol in permeate and retentate streams.

References

[1] T. Kamsuwan, C. Krutpijit, S. Praserthdam, S. Phatanasri, B. Jongsomjit, P. Praserthdam, Comparative study on the effect of different copper loading on catalytic behaviors and activity of Cu/ZnO/Al2O3 catalysts toward CO and CO2 hydrogenation, Heliyon 7 (2021) e07682. https://doi.org/https://doi.org/10.1016/j.heliyon.2021.e07682.

[2] Z.G. Duma, X. Dyosiba, J. Moma, H.W. Langmi, B. Louis, K. Parkhomenko, N.M. Musyoka, Thermocatalytic Hydrogenation of CO2 to Methanol Using Cu-ZnO Bimetallic Catalysts Supported on Metal–Organic Frameworks, Catalysts 12 (2022). https://doi.org/10.3390/catal12040401.

[3] I. Melián-Cabrera, M. López Granados, J.L.G. Fierro, Structural reversibility of a ternary CuO-ZnO-Al 2 O 3 ex hydrotalcite-containing material during wet Pd impregnation, Catal. Letters 84 (2002) 153–161.
